# Long-Term Transplant Effects of iPSC-RPE Monolayer in Immunodeficient RCS Rats

**DOI:** 10.3390/cells10112951

**Published:** 2021-10-29

**Authors:** Deepthi S. Rajendran Nair, Danhong Zhu, Ruchi Sharma, Juan Carlos Martinez Camarillo, Kapil Bharti, David R. Hinton, Mark S. Humayun, Biju B. Thomas

**Affiliations:** 1 Department of Ophthalmology, Roski Eye Institute, Keck School of Medicine, University of Southern California, Los Angeles, CA 90033, USA; deepthir@usc.edu (D.S.R.N.); juan.martinez@med.usc.edu (J.C.M.C.); humayun@med.usc.edu (M.S.H.); 2 Department of Pathology and Ophthalmology, USC Roski Eye Institute, Keck School of Medicine, University of Southern California, Los Angeles, CA 90033, USA; dzhu@usc.edu (D.Z.); dhinton@usc.edu (D.R.H.); 3 Unit on Ocular and Stem Cell Translational Research, National Eye Institute, NIH, Bethesda, MD 20892, USA; fnu.ruchi2@nih.gov (R.S.); kapil.bharti@nih.gov (K.B.); 4 USC Ginsburg Institute for Biomedical Therapeutics, University of Southern California, Los Angeles, CA 90033, USA

**Keywords:** iPSC-RPE, retinal pigment epithelium, immunodeficient RCS rat, ultrathin parylene, retinal degeneration, retinal transplantation

## Abstract

Retinal pigment epithelium (RPE) replacement therapy is evolving as a feasible approach to treat age-related macular degeneration (AMD). In many preclinical studies, RPE cells are transplanted as a cell suspension into immunosuppressed animal eyes and transplant effects have been monitored only short-term. We investigated the long-term effects of human Induced pluripotent stem-cell-derived RPE (iPSC-RPE) transplants in an immunodeficient Royal College of Surgeons (RCS) rat model, in which RPE dysfunction led to photoreceptor degeneration. iPSC-RPE cultured as a polarized monolayer on a nanoengineered ultrathin parylene C scaffold was transplanted into the subretinal space of 28-day-old immunodeficient RCS rat pups and evaluated after 1, 4, and 11 months. Assessment at early time points showed good iPSC-RPE survival. The transplants remained as a monolayer, expressed RPE-specific markers, performed phagocytic function, and contributed to vision preservation. At 11-months post-implantation, RPE survival was observed in only 50% of the eyes that were concomitant with vision preservation. Loss of RPE monolayer characteristics at the 11-month time point was associated with peri-membrane fibrosis, immune reaction through the activation of macrophages (CD 68 expression), and the transition of cell fate (expression of mesenchymal markers). The overall study outcome supports the therapeutic potential of RPE grafts despite the loss of some transplant benefits during long-term observations.

## 1. Introduction

Age-related macular degeneration (AMD), one of the most common causes of blindness in the developed world, is a degenerative disease of the retina often leading to progressive vision loss. Geographic atrophy, the advanced form of AMD, is characterized by dysfunction of retinal pigmented epithelium cells (RPEs) followed by degeneration of overlying photoreceptors leading to the loss of central vision. At present, no proven clinical treatments exist for the preservation or replacement of vulnerable RPE cells; however, RPE cell transplantation is perhaps the most obvious therapeutic option and has garnered significant interest. In the early stages of AMD, although the RPE cells are dysfunctional, surviving photoreceptors and the inner retina that transmit visual signals to the brain remain functional, rendering a realistic possibility that replacing the degenerating RPE with functional young RPE will restore vision.

Potential sources of healthy RPEs are pluripotent cells derived from embryonic [1,2,3,4] or adult cell sources [5,6,7,8,9], which are differentiated into RPE cells by employing spontaneous or directed differentiation methods. Early-phase clinical trials by various research groups used embryonic stem-cell-derived RPE (ESC-RPE) for cell replacement, which has already shown early signals of safety and potential efficacy [10,11,12,13,14]. Our team has demonstrated that human embryonic stem-cell-derived RPE (hESC-RPE) grown as a polarized monolayer on ultrathin parylene substrates can remain functional after transplantation in athymic nude rats [15] and in Royal College of Surgeons (RCS) rats [16,17], a model for RPE dysfunction. The product, termed the California Project to Cure Blindness-RPE (CPCB-RPE1), is being assessed in an FDA-approved phase1/2a clinical trial (NCT 02590692) for advanced dry AMD and exhibits promising outcomes for improving visual activity [12].

The autologous induced pluripotent stem-cell-derived RPE (iPSC-RPE) transplantation is considered more advantageous as the chance of graft rejection issues can be minimized. Recent research focuses on the generation of iPSC lines from adult cell sources, such as skin fibroblasts or peripheral blood mononuclear cells [5,6,7,8,9]. The four-year report of iPSC-RPE sheet transplant surgery for CNV (choroidal neovascularization—the wet form of AMD) in one patient has been published recently [18]. Another major step forward is the allogeneic transplantation of off-the-shelf available iPSC-RPE. Due to concerns regarding possible oncogenic mutations in cell preparation, the attention is now focused on personalized screening for mutations and the development of autologous iPSC-RPE therapies including HLA matching [19,20]. A study by Sugita et al. [19] aimed at examining the safety of six-loci HLA-matched allogeneic iPSC-RPE transplantation under local steroids. RPE cells grafted as a suspension into the patient’s subretinal space survived in all five cases for more than one year [19]. These observations suggest that it is possible to manage the survival of iPSC-RPE, under immunosuppression. However, regenerative medicine is still in its infancy and the cells may behave differently in each individual. In the first iPSC-RPE transplantation clinical study [14,21], three aberrations in the deoxyribonucleic acid (DNA) copy number (deletions) were observed in the cell preparation of the second patient, which caused the study to end due to possible adverse effects.

Existing evidence indicates that the delivery of cells as a suspension may not consistently develop into a monolayer of RPE and that their long-term survival rate will be low compared to RPE cells transplanted as a monolayer [15]. In many preclinical studies for geographic atrophy, the iPSC-RPEs were delivered into the subretinal space as a bolus injection [22,23,24,25] and the animals were monitored for survival under immunosuppression for only a short period. In other studies, iPSC-RPEs transplanted as a monolayer and maintained under immunosuppression regimes were followed for up to 5 months [9,18]. Published data suggest that a confluent polarized monolayer of iPSC-RPEs transplanted as a patch rather than as a cell suspension can perform several basic functions of RPEs including phagocytosis of photoreceptor outer segments, the renewal of visual pigment, and the transport of metabolites [9,14]

Although transplantation of iPSC-RPE cells to replace the diseased RPE has been tested by several groups through preclinical studies and there are preliminary reports of ongoing preclinical studies, no major attempt has been made to perform an in-depth analysis of the long-term viability and fate of the transplanted RPE. Based on previous reports from our group [17], the progressive deterioration of visual function after the transplantation of ESC- RPE was evident during long-term observations. Monitoring cell survival and assessing the long-term functional benefits of transplants are significant since the transplanted cells are exposed to a progressively degenerating environment and there may be immunological factors that can cause adverse effects.

In preclinical studies of RPE transplantation, one of the major factors that influence the long-term benefits is the immune reaction and associated xenograft rejection. Sharma et al. [9] showed 70% survival of subretinally transplanted human iPSC-RPE (hiPSC-RPE) cells up to 2.5 months post-implantation in immunosuppressed rodents. In the above study, systemic and resident innate immune responses in animal models were suppressed by using prednisone, doxycycline, and minocycline whereas the adaptive immune responses were suppressed using tacrolimus and sirolimus [9]. Del Priore et al., in 2003 [26], demonstrated ‘triple systemic’ therapy with anti-inflammatory antibiotics to increase the survival of grafted RPE at four weeks post-implantation. However, based on a previous report, immunosuppressants can alter the visual function in RCS rats with depressed scores on behavioral and electrophysiological testing [27]. Hence, to minimize the complications associated with immunosuppressants, we used a newly developed immunodeficient RCS rat model characterized by an absence of T cells and a lack of natural cell-mediated cytotoxicity [28]. The iPSC-RPE cells grown as a polarized monolayer on ultrathin perylene substrates were transplanted into the subretinal space of immunodeficient RCS rats. The transplant effects were assessed at various post-implantation time points (1 to 11 months after transplantation).

## 2. Materials and Methods

### 2.1. Human Pluripotent Stem Cells Generated from iPSCs

iPSC-RPE (frozen, passage 2 cells) generated from iPSCs, reprogrammed from healthy adult fibroblasts, was obtained from Dr. Kapil Bharti, Unit on Ocular and Stem Cell Translational Research, National Eye Institute, NIH, Bethesda, USA [29]. Briefly, hiPSC were seeded at 20,000 cells per cm^2^ on Matrigel and grown in mTeSR1 in a 10% CO_2_/5% O_2_ incubator for 5 days. Afterward, they were transferred to a 5% CO_2_/20% O_2_ incubator and cultured for 5 additional days. At this point, the culture medium was switched to a differentiation medium (DM) [29]. After 10–15 days, cells were maintained in DM for 3 more weeks and then switched to an RPE maintenance medium (RPEM) [29]. Differentiated cells were dissociated in Accumax (Sigma, Saint Louis, MO, USA), plated at 250–300,000 cells per cm^2^, and grown in RPEM. The passage 3 cells were used for transplantation experiments. These cells are extensively characterized for clinical applications in Dr. Bharti’s lab [9,30,31].

### 2.2. Preparation of Polarized hESC-RPE Implant on Parylene Membranes

Ultrathin parylene membranes (0.3 μm thickness supported on a 6.0 μm-thick mesh frame) made from parylene C were specially designed for implantation on rat retinas (1.0 × 0.4 mm) [15,17] and used successfully for culturing iPSC-RPEs. These ultrathin membranes were coated with Matrigel (AMS Biotechnology, Frankfurt, Germany) and seeded with iPSC-RPE based on our previously established protocol [17]. The cells were grown to confluence for approximately 4 weeks before implantation. The final density of each implant was kept as approximately 2700 cells/membrane [15].

### 2.3. Immunostaining of iPSC-RPE on Parylene Membrane

iPSC-RPE cells grown on Matrigel-coated parylene were stained for RPE specific markers zonula occludens protein 1 (ZO-1), and RPE65, based on established protocols [17]. Stained cells were mounted with an anti-fading mounting medium (Invitrogen) and images were captured by confocal microscopy (FV1000 Confocal Microscope, Olympus, Centre Valley, PA, USA).

### 2.4. Animals

The Royal College of Surgeons (RCS) rat is an established model of retinal degeneration, which has been mainly used for studying photoreceptor rescue with treatment at the age of 3–4 weeks. These rats develop a fully functional visual system, which degenerates secondarily due to their dysfunctional RPE (MertK mutation), resulting in the loss of most photoreceptors at the age of 3 months. Immunodeficient RCS rats were produced from a cross between female homozygous RCS (RCS-p+/RCS-p+) and male athymic nude rats (Hsd: RH-Foxn1mu, a mutation in the foxn1 gene; no T cells) as described previously [28]. All rats were maintained in an aseptic and temperature-controlled environment. All animals were included in accordance with the Association for Research in Vision and Ophthalmology (ARVO) statement for the use of animals in research, and the Institutional Animal Care and Use Committee (IACUC) of USC.

### 2.5. Surgical Procedure

Animals underwent surgery at postnatal day (P) 28. Anesthesia was induced by intraperitoneal injection of ketamine (37.5 mg/kg) and xylazine (5 mg/kg). Only the left eyes were used for transplantation surgeries. All surgeries were performed by the same surgeon. Topical anesthesia was administered with a 0.5% proparacaine hydrochloride ophthalmic solution (Akorn, Inc., Lake Forest, IL, USA). Pupils were dilated using ophthalmic solutions of 2.5% phenylephrine hydrochloride and 0.5% tropicamide (Akorn, Inc., Lake Forest, IL, USA). Once the conjunctiva is removed, a scleral incision was performed in the temporal superior quadrant followed by an anterior chamber paracentesis to reduce intraocular pressure. A small incision (approximately 0.8–1.0 mm) was cut transsclerally at the temporal equator of the eye until the choroid was exposed with the help of a 32-gauge needle, and a 5 μL balanced salt solution (Alcon Laboratories, Inc., Fort Worth, TX, USA) was injected to create a local retinal detachment. The implant held by forceps was introduced through the sub scleral space into the subretinal bleb. Clinical assessment as well as retinal imaging by optical coherence tomography (OCT) using a Spectralis HRA + OCT device (Heidelberg Engineering, Heildeberg, Germany) were performed to confirm the placement of the implant. The rats were then allowed to recover from anesthesia in a thermal care incubator. Animals with surgical complications such as excessive bleeding, perforation of the retina, and implant delivery into the vitreous were immediately excluded from the study. Based on OCT images, 15 animals were selected for short-term experiments (1-month and 4-month study group) and 15 animals were selected for the 11-month study group.

### 2.6. Histopathology

Cohorts of rats were euthanized by intracardiac injection of euthasol (Virbac AH, Inc., Fort Worth, TX, USA) at 1, 4-, and 11-months post-surgery, and eyes were processed for histology. Contralateral eyes were considered as controls. Whole eyes were fixed in Davidson’s solution overnight, and the cornea and lens were removed. Finally, the eye cups were embedded in paraffin and processed for sectioning (5-μm sections). Groups of consecutive slides were stained with hematoxylin and eosin (HE) for light microscopy. The HE-stained slides were scanned and photographed using an Aperio Scanscope CS (Aperio Technologies, INL., Vista, CA, USA) microscope. Histological sections of cell-seeded membranes were evaluated to assess iPSC-RPE survival. The surgical placement was considered acceptable if more than 70% of the implant was located inside the subretinal area. Transplant survival was confirmed only if iPSC-RPE were observed in at least three consecutive sections based on light microscopy and immunostaining evaluations. Cell migration or dead cell aggregation was considered when pigmented cells or cell clumps were seen adjacent to the substrate and confirmed by immunohistochemistry. If no human/RPE marker was found, the specimen was considered as non-surviving RPE clumps. The outer nuclear layer (ONL) integrity was evaluated for photoreceptor preservation in the transplanted area. Cellular reaction around the implants, observed by light microscopy, was assessed for the presence of macrophages or the expression of glial cells. Adjacent sections of the implanted eye were processed for immunohistochemical analysis using the following antibodies as needed: Human-specific cell surface marker (anti-TRA-1-85), a marker of differentiated RPE cells (anti-RPE65), a macrophage marker (anti-CD68), an astrocyte/Müller cell marker (anti-GFAP), mesenchymal markers (α Smooth muscle actin and Vimentin), photoreceptor phagocytosis marker (Rhodopsin), and RPE binding protein (RBP1).

Details of the antibodies used are included in Table 1. For immunostaining, all slides were deparaffinized, rehydrated, and antigen retrieved (sodium citrate, pH 6.0). After staining, the slides were mounted with fluorescent-enhanced mounting medium with 4′,6-diamidino-2-phenylindole (DAPI) (Vector Laboratory, Burlingame, CA, USA). Images were taken using the Ultra viewer ERS dual-spinning disk confocal microscope (PerkinElmer, Waltham MA, USA) equipped with a C-Apochromat (Carl Zeiss, Thornwood, NY, USA) ×10 high dry lens, a C-Apochromat ×40 water immersion lens NA 1.2, an electron multiplier charge-coupled device cooled digital camera (Hamamatsu Orce_ERCC 12-bit camera]; PerkinElmer, Waltham, MA, USA) or by using a Keyence BZX-800 microscope. Images were captured and processed using PerkinElmer Velocity imaging software. 

### 2.7. Superior Colliculus Electrophysiology

Electrophysiological mapping of the superior colliculus (SC) was performed at approximately 11-months post-surgery based on an established protocol followed in our laboratory [7,17,28]. Based on OCT screening, 8 rats (8/15) were selected for SC experiments. Rats dark-adapted overnight were anesthetized by an intraperitoneal injection of xylazine/ketamine. The gas-inhalant anesthetic (1–2.0% isoflurane) was administered via an anesthetic mask (Stoelting Company, Wood Dale, IL, USA). Rats were mounted in a stereotactic apparatus; a craniotomy was performed, and the SC was exposed. Multi-unit visual responses were recorded extracellularly from the superficial laminae of the SC using custom-made tungsten microelectrodes. For SC mapping, the responses were recorded from approximately 30 different SC locations. At each recording location, approximately 10 presentations of a full-field strobe flash (1300 cd m22, Grass model PS 33 Photic stimulator, W. Warwick, RI, USA), positioned 30 cm in front of the rat’s eye, were delivered to the contralateral eye. An interstimulus interval of 5 s was used. The neural activities were recorded using a digital data acquisition system (Power lab; ADI Instruments, Mountain View, CA, USA) 100 milliseconds before and 500 milliseconds after the onset of the stimulus. All responses at each site were averaged. Blank trials, in which the illumination of the eye was blocked with an opaque filter, were also recorded at each site.

### 2.8. Optokinetic Testing

Optokinetic (OKN) testing was performed at 4 months and 11 months post transplantation using a previously described protocol [17]. To record OKN responses, two tablet screens were used to display the OKN stimuli consisting of high-contrast black and white stripes generated using ‘OKN Stripes Visualization Web Application’, a freely available software (http://mdds.nyc/okn-stripes-visualization, accessed on 12 April 2021). A clear plexiglass holder was used to restrain the rat and keep its head continuously exposed to the tablet screen. A micro camera attached to the top of the rat holder recorded the headtracking responses during clockwise (1 min) and anticlockwise (1 min) stripe rotations. Visual acuity was tested by changing the stripe width at decrements of 0.5. Video recordings were evaluated to compute the head-tracking scores by two separate investigators who were both masked to the experimental condition. The OKN responses at various spatial frequencies were assessed based on the presence or absence of clear head-tracking and based on the duration of head-tracking.

### 2.9. Statistical Analysis

Statistical comparisons were made using GraphPad Prism software (GraphPad Software Inc., La Jolla, CA, USA). The Paired *t*-test was used for analyzing the OKN data. The remaining data were analyzed using Student’s *t*-test or by the Analysis of Variance (ANOVA) followed by the appropriate post hoc test. For all comparisons, the significance level was determined at *p* < 0.05.

## 3. Results

### 3.1. Human iPSC-RPE Cells Can Grow as a Polarized Monolayer over Ultrathin Parylene Membrane and Demonstrate High-Purity and RPE Marker Expression

**Figure 1:**
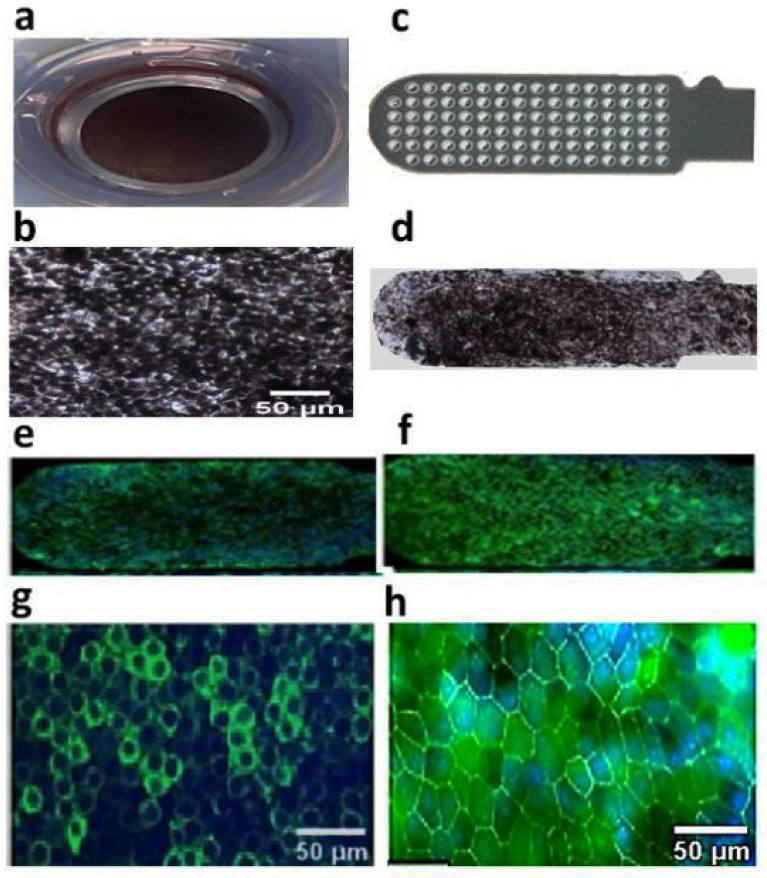
iPSC-RPE grown as polarized monolayer over parylene substrate. (**a**) iPSC-RPE polarized monolayer cultured on 24-well transwell insert, (**b**) enlarged view of iPSC-RPE monolayer, (**c**) ultrathin parylene membrane without cells, (**d**) iPSC-RPE grown as polarized monolayer on parylene membrane, low magnification (10×) image showing the whole implant stained for **(e)** RPE 65 (**f**) ZO-1 expression, enlarged view of expression of (**g**) RPE 65 and (**h**) ZO-1 on parylene membrane.

iPSC-RPE cells (Figure 1a,b) cultured on a Matrigel-coated ultrathin parylene substrate were expanded as a polarized confluent monolayer (Figure 1d) and expressed RPE 65 and ZO-1 as evidenced by immunocytochemistry (Figure 1e–h). This study demonstrated that iPSC-RPE can be grown as a polarized monolayer on ultrathin parylene, similar to our previous hESC-RPE implants [17].

### 3.2. iPSC-RPE Implant Survival and Functionality Assessed by Short-Term in Vivo Experiments in Immunodeficient RCS Rats (1- and 4-Month Study)

**Figure 2:**
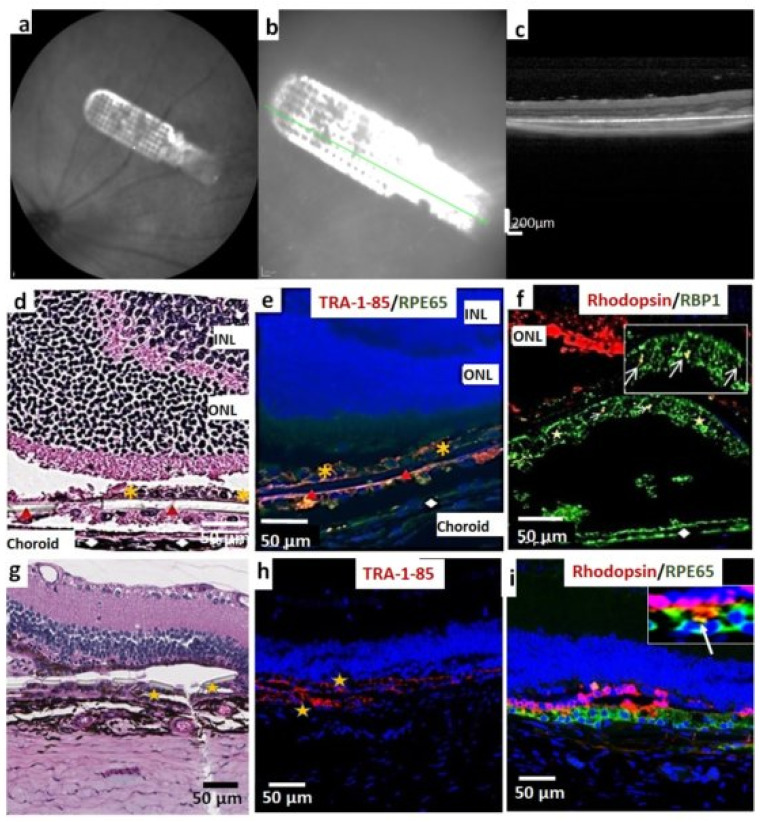
Short-term assessment of iPSC-RPE implant survival and functionality, in immunodeficient RCS rats. (**a**) iPSC-RPE implants observed during fundus examination of immunodeficient RCS rats at 1 month post-implantation, (**b**) enlarged view, (**c**) vertical OCT b-scan image through the transplant area, (**d**) HE image showing subretinal implant placement. The choroidal layer that appears to be separated from the remaining retina is considered a histologic artifact. Yellow asterisk indicates iPSC-RPE cells; Red arrow heads indicate the parylene membrane (**e**) transplant is identified by TRA-1-85 (human specific marker, red) and RPE65 (green) expression; Red arrowhead indicates the parylene substrate; white rhombus represents endogenous rat RPE, yellow asterisk indicates RPE on Parylene membrane (**f**) Rhodopsin (red) and retinol-binding protein (RBP1, green) staining to demonstrate that implanted iPSC-RPE can phagocytose photoreceptor outer segments (white arrows). Inset is a higher magnification of the above area. Red arrowhead indicates the parylene substrate; white rhombus represents endogenous rat RPE; (**g**) HE image showing subretinal implant 4 months after transplantation; (**h**) transplant at 4 months is identified by TRA-1-85 (human specific marker, red) (**i**) Rhodopsin (red) and RPE 65 (green) staining were used to show that implanted iPSC-RPE can phagocytose photoreceptor outer segments at 4 months after transplantation. Inset is a higher magnification of the transplant area indicating phagocytosis (white arrow).

After the transplantation surgery, OCT imaging was performed to screen the animals for proper implant placement (Figure 2a–c). Animals with the implant placed as a flat sheet adjacent to the Bruch’s membrane were selected for further analysis. Histological analysis at 1-month post-implantation showed the presence of a well-pigmented, intact iPSC-RPE cell layer attached to the parylene substrate in all transplanted eyes. No major signs of inflammation were observed in any of the implanted animals. A majority of the retinas (92.0%) maintained the basic retinal architecture without noticeable structural changes. The histological analysis revealed that transplanted cells survived very well, evidenced by TRA-1-85 (human specific marker) expression and retinol-binding protein expression (Figure 2e,f). Transplants in which iPSC-RPEs were present on the lower surface of the parylene membrane also showed good survival (Figure 2e). At the 1-month time point, the expression of CD68 (macrophage marker) and GFAP (retinal glial marker) was not observed in the transplant area (Supplementary Figure S1) or in the area outside the transplant (data not shown). The cells retained RPE 65 expression and human marker expression (Tra-1-85) without any evidence of mesenchymal marker expression (vimentin and α-smooth muscle actin, see Supplementary Figure S2). Good survival of iPSC-RPE implants was also observed at 4 months post-implantation (Figure 2g–i). Based on rhodopsin staining, the surviving cells performed a phagocytic function (Figure 2i). The absence of immunological markers comparable to the control group indicate the absence of detectable chronic inflammation induced by xenografts.

### 3.3. In Vivo Assessment of Long-Term Transplant Effects in Immunodeficient RCS Rats (11 Month Study)

**Figure 3:**
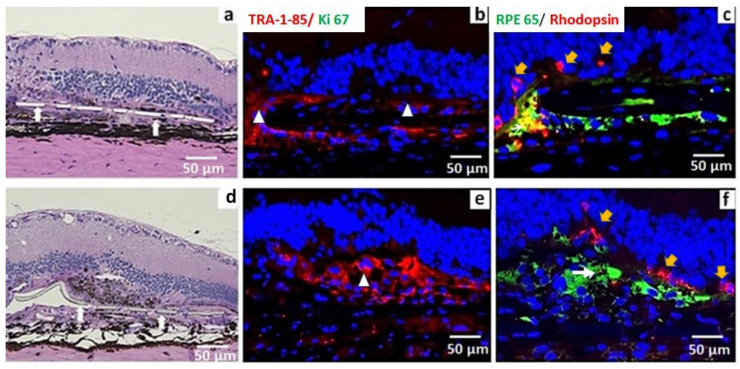
Representative HE and immunostaining images of immunodeficient RCS rats implanted with iPSC-RPE monolayer assessed at 11 months post implantation. Large white arrows (**a**,**d**) point to the parylene membrane. (**a**–**c**) Retina containing iPSC-RPE monolayer, (**d**–**f**) retina with iPSC-RPE appeared as multiple cell layers or cell clumps. TRA-1-85 white triangle in (**b**,**e**) and RPE65 expression were used for identifying iPSC-RPE. Absence of Ki67 expression indicates absence of proliferative cells (**b**,**e**). Rhodopsin immunostaining is used to identify photoreceptor survival yellow arrows in figure (**c**,**f**). Rhodopsin-containing phagosomes are found in the transplanted iPSC-RPE denoted by white arrows (**c**,**f**). Phagocytic activities were prominent in eyes in which monolayer structure was better preserved (**c**).

Evaluation of histology images from serial sections at 11 months post-implantation showed the presence of transplanted RPE in seven eyes (7/15). Out of these seven eyes, four eyes retained an intact RPE monolayer structure. Immunostaining showed rhodopsin-containing phagosomes in the transplanted RPE. This was more prominent in eyes in which better preservation of the iPSC-RPE monolayer structure was observed (Figure 3c). In the remaining three eyes, the cells appeared as clumps (Figure 3e,f) out of which only two eyes retained RPE65 expression. There was no Ki 67 expression in the implanted areas, suggesting an absence of proliferative cells. Photoreceptor outer nuclear layer (ONL) preservation was evident in almost all eyes in which strong RPE65 expression was noticed (Figure 3c,f). A complete loss of transplanted cells was noticed in eight (8/15) eyes.

**Figure 4:**
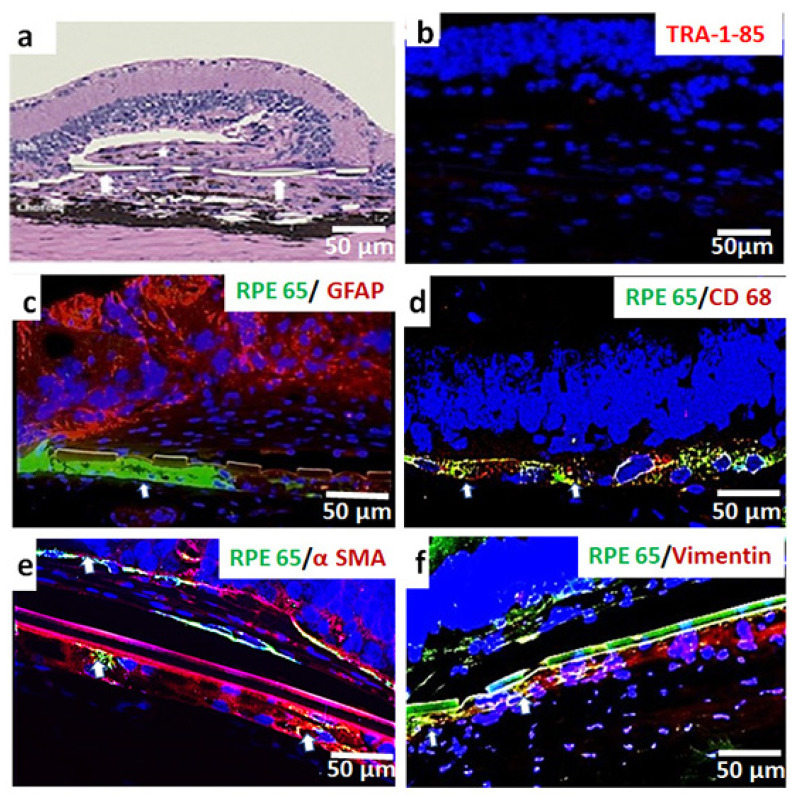
Representative HE and immunostaining images of immunodeficient RCS rat retinas implanted with iPSC-RPE monolayer assessed at 11 months post-implantation. Presence of fibrosis, immunoreactivity, and epithelial–mesenchymal transition (EMT) was assessed. (**a**) Retina with no surviving iPSC-RPE showing signs of inflammation and peri-membrane fibrosis indicated by white asterisk. (**b**) Absence of TRA-I-85 staining. (**c**) Retinas showing RPE65 expressing iPSC-RPE cells (white arrows) labelled for GFAP (glial cells), (**d**) CD68 (macrophages/microglia), (**e**) expression of classical mesenchymal markers α smooth muscle actin α SMA and vimentin. (**f**) Images in which iPSC-RPE monolayer appears to be present below the parylene membrane are either due to orientation difference in the implant placement or due to the survival of the RPE on the lower side of the parylene membrane.

ONL preservation was not observed in the eyes in which iPSC-RPE survival was absent. The presence of fibrosis was noticed in the majority of the above eyes (Figure 4a,b). A summary of the histological result of the 11-month post-implantation study is given in Table 2.

The expression of CD68 and GFAP was used to analyze inflammatory and glial reactions to donor tissues. GFAP was strongly expressed in the ganglion cell layer, the inner nuclear layer, and the choroid area, but was absent in the transplant area (Figure 4c). CD68 positivity observed in some of the implanted eyes suggests inflammatory reactions associated with transplantation (Figure 4d). To validate the cell loss associated with the loss of tight junctions and consequent loss of cell–cell contact and cell–matrix contact, the tissue was tested for classic EMT markers—α smooth muscle actin (α SMA) and vimentin. Interestingly, in the implants in which RPE expression was absent or feeble, there was a strong expression of α smooth muscle actin (Figure 4e), and vimentin (Figure 4f) was also observed.

### 3.4. Preservation of Low Light Level Visual Responses in the Superior Colliculus (SC) of iPSC-RPE-Implanted Rats at 11-Month Post-Implantation

**Figure 5:**
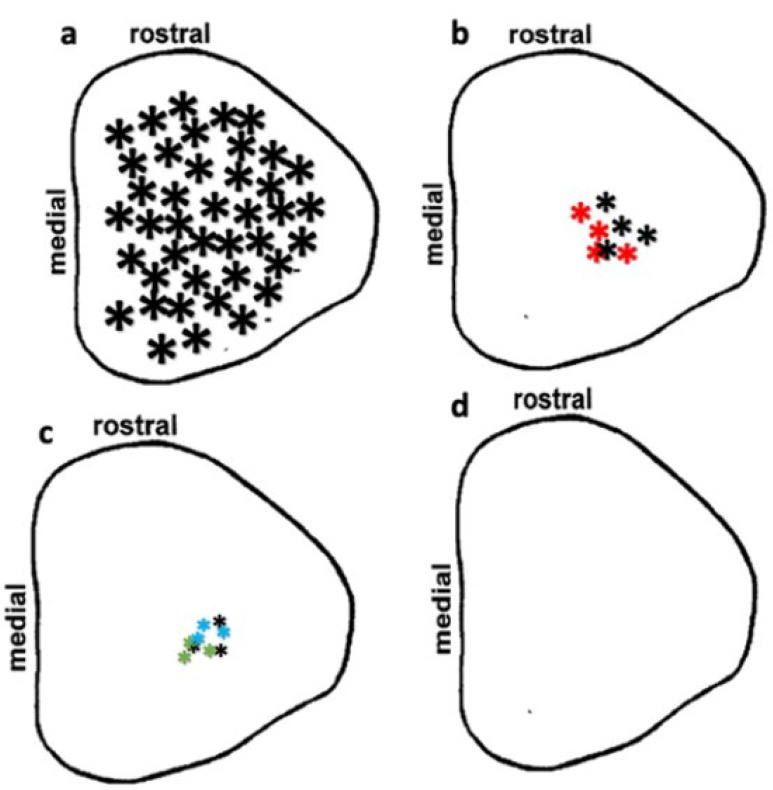
Visual activities recorded from the SC of 11-month-old immunodeficient RCS rats. Map properties of SC-evoked responses from individual rats are represented by colored asterisks. Larger asterisks show higher light sensitivity in the SC. (**a**) Age-matched normal rat. (**b**) * Rat # 6005 and * Rat # 6012. (**c**) * Rat # 6001, * **Rat # 6006**, and * Rat # 6011. Based on morphological examination, all these rats showed surviving iPSC-RPE in the retina (see Table 2). (**d**) No light-evoked visual activity was observed in the remaining transplanted rats and age-matched control RD rats.

iPSC-RPE-implanted immunodeficient RCS rats were subjected to SC luminance threshold mapping (Figure 5). Electrophysiological mapping of the SC allowed for correlation of the response area in the SC with the location of the implant placement in the eye based on the established retinocollicular map properties [32]. Age-matched normal Long Evans (LE) rats showed visual activity from all over the SC (Figure 5a). Among transplanted rats, visual preservation was observed in only five rats (5/8).

Visually evoked activities in these rats were observed only in a small SC area corresponding to the implant placement in the eye. Visual activities were robust (higher light sensitivity) in two of the above rats (Figure 5b) whereas only weak visual activity (lower light sensitivity) was recorded in the remaining three rats (Figure 5c). No light-evoked visual activity was observed in the SC of age-matched non-transplanted rats (Figure 5d). All five rats that showed SC visual activity showed a presence of transplanted RPE in their eyes (Table 2). No correlation was observed between the light-sensitivity threshold and the degree of transplant survival.

### 3.5. Optokinetic (OKN) Responses in iPSC-RPE-Implanted Rats

**Figure 6:**
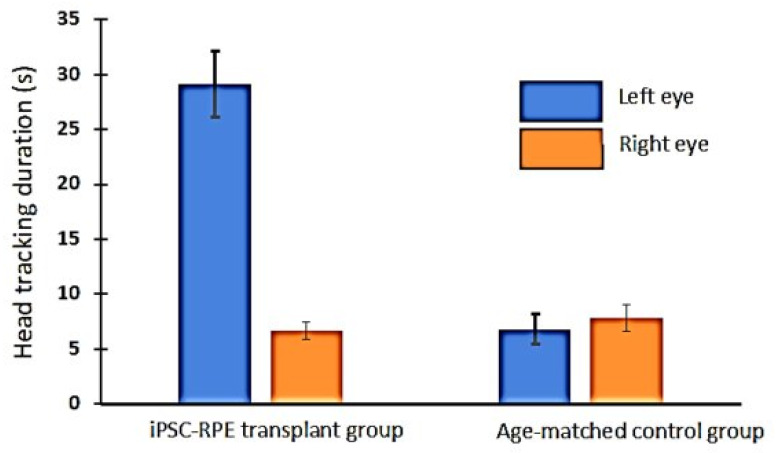
OKN testing data based on the duration of head-tracking recorded from 4-month-old immunodeficient RCS rats (±SE). The data show improved head-tracking response in the iPSC-RPE transplanted left eyes (*n* = 12) compared to the non-transplanted eyes and age-matched control rats (*n* = 5).

Based on OKN data, visual improvement in the iPSC-RPE-implanted eyes was observed at 4 months post-transplantation (Figure 6). However, when tested at the 11-month time point, no measurable OKN responses were observed in any of the iPSC-RPE-implanted rats (data not shown).

## 4. Discussion

Ongoing multicentral clinical studies have proven that stem-cell-derived RPE transplantation is a practical option to restore failing vision in retinal dystrophies [12,13,19,24]. Previous animal studies and pilot data from our Phase l/ll clinical studies demonstrated the feasibility of using ultrathin parylene as a bio membrane for hESC-RPE growth and subretinal implantation [12,15,17,33]. Compared to hESC-RPE, using iPSC-derived RPE is considered more advantageous due to the possibility of generating a sufficient number of autologous RPE cells that strongly resemble primary human RPEs and its potential to minimize issues associated with immune rejection. Based on this, the present study evaluated the long-term benefits of polarized iPSC-RPE cells grown on an ultrathin parylene membrane that can act as an artificial Bruch’s membrane. The ability of such implants to support transplant survival and viability of the host photoreceptors to preserve visual function is demonstrated in a new immunodeficient RCS rat model. 

The majority of the previous iPSC-RPE transplantation studies were conducted in immunosuppressed animal models, and assessments were made for a short duration only, which may not be sufficient to extrapolate into long-term implications [9,34,35,36]. Hence, in the present study, transplant effects were analyzed in a new immunodeficient RCS rat model, and assessments were conducted up to one year after implantation. The results from this study demonstrated the safety and potential bioactivity of the iPSC-RPE implant both during the short-term (1–4 months) investigation and long-term investigation (11-month study). Based on histology assessments, good coverage of the implanted iPSC-RPE on the parylene membrane was observed in the majority of eyes up to 4 months post-implantation along with improvement in visual function confirmed by OKN testing. In our long-term studies (11 months post-transplantation), iPSC-RPE survival and phagocytic function were only observed in less than 50% of the transplanted rats (7/15). In another report, a loss of transplanted iPSC- RPE in RCS rats, immunosuppressed by oral administration of cyclosporin, was observed at 13 weeks post-transplantation [36]. Based on the available data, the loss of transplanted RPE over the course of time and alteration in the monolayer structure can be related to the immune reaction to xenografts [19,37,38].

Previous studies have shown that the survival and integration of transplanted ESC-RPEs in the pathologic environment of a diseased retina is challenging due to it being prone to attack by macrophages [15]. CD 68 expression has been reported in studies using hESC-RPE transplantation and hESC-RPE cell suspension injections in immunosuppressed RCS rats [15,38,39]. In the present investigation, we used immunodeficient RCS rats to reduce immunological issues. However, signs of inflammation and microglia activation have been previously reported in immunodeficient RCS rats [28]. Hence, we used CD68 as a marker for assessing reactive microglia in the transplanted eyes. In our long-term studies, some CD 68 expression was observed in the implants and in areas adjacent to them (Figure 5d). However, this phenomenon was not observed at the early timepoint (1-month post-implantation, see Supplementary Figure S1). This suggests that reactive microglia/macrophages can play a role in the loss of transplanted RPEs at a later time point. In contrast to the above reports, a recent study by Zhu et.al suggested that an iPSC-PRE cell suspension injection can lower the microglial activation (CD68 expression) in rd10 mice [34]. The discrepancies in the study outcomes may be related to the differences in the animal models used, the time points in which CD68 staining was conducted, and the cell types used for transplantation experiments.

Glial fibrillary acid protein (GFAP) expression, which is known to occur in response to retinal injuries [40], can be also suggested to play a role in transplant loss in the 11-month study group. However, GFAP expression in the above study group was found to be mostly adjacent to the inner nuclear region, choroid area, and ganglionic layer, which is far from the implant area. Since this GFAP expression pattern is comparable to that of the non-transplanted control eyes [41], the presence of GFAP cannot be correlated to the loss of transplanted iPSC-RPEs.

In some of our transplanted eyes, the iPSC-RPEs developed into cell clumps on the surface of the parylene membrane (Figure 3d). Previous studies suggested that when the RPE transplant fails to establish a monolayer and form cell clumps, its survival will be poor and the cells will not be capable of performing normal RPE functions [42,43]. Based on this finding, we suggest that implants may need to be microscopically examined for potential signs of clumping prior to subretinal implantation.

The cell clump formation observed in about 20% of the implanted eyes (long-term study group) might have occurred even after transplantation due to cell migration. Cell migration is mainly attributed to the loss of RPE tight junctions [44]. This can lead to a loss of cell-to-cell contact and anchorage dependence, which are critical for RPE survival and functionality [45]. Emerging evidence demonstrates that RPE cells can be less differentiated and undergo the epithelial–mesenchymal transition (EMT) and enhanced migration in retinal degenerative diseases, including macular degenerations and proliferative vitreoretinopathy [46,47,48,49]. Such a transition is also reported in higher-passage RPEs during in vitro observations [50,51]. Immunostaining of transplanted eyes from the 11-month study group revealed the presence of two classic mesenchymal cell markers, namely α SMA and vimentin, especially in areas of the parylene membrane where a loss of RPE expression was noticed. The transition to a mesenchymal fate may cause a loss of tight junctions and reduced cell adherence to the parylene membrane that can lead to a fibroblastic phenotype [44,45]. According to Zhou et al. [52], RPE cells retain the reprogramming capacity to move along a continuum between polarized epithelial cells and mesenchymal cells. This shift towards a mesenchymal phenotype can be defined as RPE dysfunction [52]. This change of RPE characteristics can cause senescence/fibrosis, eventually resulting in a loss of transplanted cells. In our transplanted rats, the expression of EMT markers was not evident at the earlier time point (1-month study group) when RPE survival was more robust (Supplementary Figure S2). Further studies are needed to identify the exact time point at which the EMT markers are expressed to determine whether changes in the iPSC-RPEs take place only in the long-term post-implantation period.

The RCS retina is widely known for its acute reactions to surgical interventions. Surgical trauma in rat eyes of a severe nature as a result of their small size can lead to increased tissue reactions in the implanted area. In support of this, mild inflammation and peri-membrane fibrosis were visible around the majority of the implants, in which the RPE monolayer was lost. It may be noted that, although the immunodeficient RCS rat is T-cell deficient, they possess bone-marrow-dependent B cells and natural killer (NK) cells. All of the above factors can contribute to the loss of transplanted iPSC-RPE cells.

In the long-term study group (11-month post-implantation), the visual functional preservation (based on SC electrophysiology) was correlated to the survival of the transplanted iPSC-RPE. However, no considerable OKN visual activities were observed in these rats at this time point. Previously, in hESC-RPE-implanted immunosuppressed RCS rats, a progressive loss of OKN responses has been reported [17]. Improved OKN visual activities observed at an earlier time point (4 month) may be attributed to residual photoreceptors present in the RCS retina. When the photoreceptor degeneration is more advanced, the transplant benefit can be limited to a very small area of the retina and its contribution may not be strong enough to evoke measurable head-tracking activities.

In conclusion, the present study demonstrated the survival and functionality of iPSC-RPE transplanted as a polarized monolayer on a non-degradable substrate containing similarities to an artificial Bruch’s membrane. The transplant benefits are higher during the earlier post-implantation period. Progressive deterioration of the transplant benefits observed in this study was correlated with the loss of transplanted iPSC-RPEs. The immune reactions and subretinal fibrosis can be considered the major causes of the loss of transplanted iPSC-RPE. From a clinical perspective, many of these adverse effects can be less severe in humans due to the differences in the eye architecture, surgical procedures, and the nature of the disease microenvironment. Moreover, in human eyes, easy application of target-specific and effective immune suppressants can help to reduce potential immunological reactions.

## Figures and Tables

**Table 1:** List of antibodies used for immunostaining.

Antibodies	Purpose	Manufacturer	Catalog No	Dilution
TRA-1-85	Human marker	R&D Systems, Minneapolis, MN, USA	MAB3195	1:100
RPE65	RPE marker	Abcam	Ab231782	1:200
Rhodopsin	Rods	Abcam	Ab3267	1:100
CD68	Microglia	Abcam	ab201340	1:300
Vimentin	Mesenchymal marker	Abcam	ab137321	1:300
GFAP	Reactive glial cells	Invitrogen	MA5-12023	1:500
α Smooth muscle actin	Mesenchymal marker	Abcam	ab5694	1:250
Goat anti-mouse IgG conjugated with Rhodamine	Secondary antibody	Jackson Immuno Research, West Grove, PA, USA	115-025-146	1:500
Goat anti-rabbit IgG conjugated with FITC	Secondary antibody	Abcam	Ab150081	1:500
Ki67	Proliferation marker	Abcam	Ab16667	1:500
Donkey Anti-Mouse lgG H&L	Secondary Antibody	Abcam	Ab7003	1:500
Donkey Anti-Rabbit lgG H&L	Secondary Antibody	Abcam	Ab150063	1:500

**Table 2:** Summary of the histological result for iPSC-RPE implantation in immunodeficient RCS rats (11-month post implantation).

iPSC-RPE Implant Status			RPE65			Phagocytosis			Fibrosis/inflammation		
No cells or cells died	Presence of intact monolayer	Cells developed into clumps, no intact monolayer	++	+	−	++	+	−	++	+	−
8	4	3	4	2	9	0	4	11	2	4	9

## Data Availability

The data presented in this study are available on request from the corresponding author.

## Supplementary Materials

The following are available online at https://www.mdpi.com/article/10.3390/cells10112951/s1, Figure S1: Representative images of immunodeficient RCS rat retinas implanted with iPSC-RPE monolayer (shown by RPE65 expression) cultured on a parylene membrane assessed at 1-month post implantation, Additional labelling of the retina sections was performed for expression of (**a**) CD68 (macrophages/microglia) and (**b**) GFAP (glial cells), Figure S2: Representative images of immunodeficient RCS rat retinas implanted with iPSC-RPE monolayer (shown by RPE65 expression) cultured on a parylene membrane assessed at 1-month post-implantation. Additional labelling of the retina sections was performed for expression of classical mesenchymal markers (**a**) vimentin and (**b**) α smooth muscle actin (α SMA).
