# Supplementary material for: Long-Term Transplant Effects of iPSC-RPE Monolayer in Immunodeficient RCS Rats
Source: Cells. 2021 Oct 29;10(11):2951. doi: 10.3390/cells10112951 (PMC8616297; doi:10.3390/cells10112951)
Supplement: Supplementary file 1 [file cells-10-02951-s001.zip › cells-1406514-supplementary.pdf]

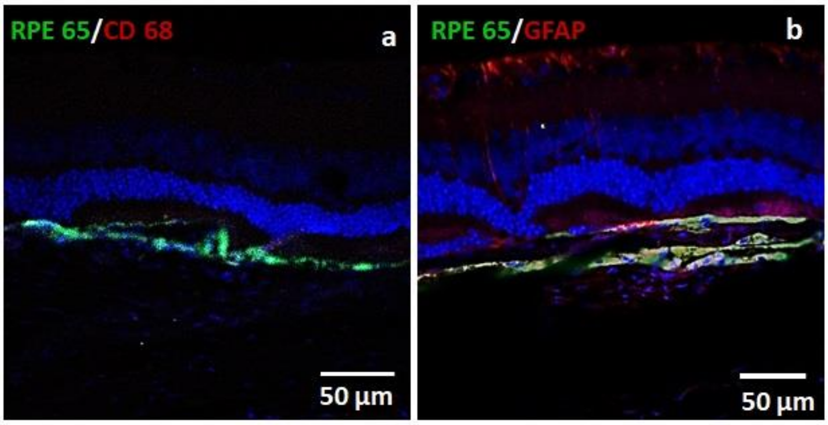

Figure S1. Representative images of immunodeficient RCS rat retinas implanted with the iPSC-RPE monolayer (shown by RPE65 expression) cultured on a parylene membrane assessed at 1-month post-implantation. Additional labelling of the retina sections was performed for expression of (**a**) CD68 (macrophages/microglia) and (**b**) GFAP (glial cells).

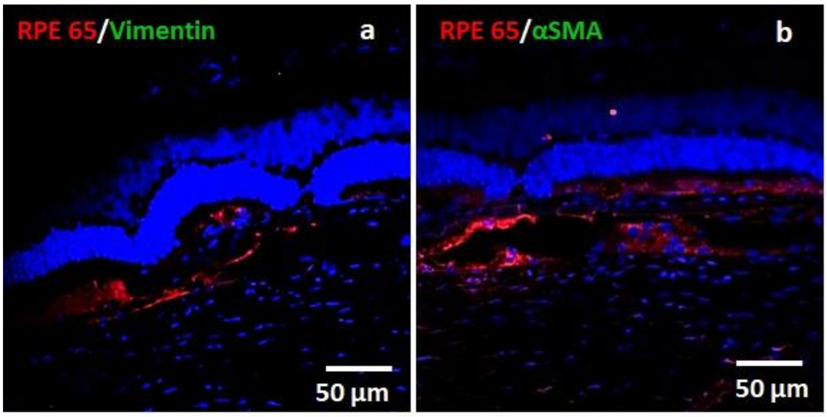

Figure S2. Representative images of immunodeficient RCS rat retinas implanted with iPSC-RPE monolayer (shown by RPE65 expression) cultured on a parylene membrane assessed at 1-month post-implantation. Additional labelling of the retina sections was performed for expression of classical mesenchymal markers (**a**) vimentin and (**b**) α smooth muscle actin (α SMA).
